# Understanding Anionic Hyperporphyrins: TDDFT Calculations on Peripherally Deprotonated *meso*-Tetrakis(4-hydroxyphenyl)porphyrin

**DOI:** 10.1021/acs.jpca.4c07216

**Published:** 2025-01-30

**Authors:** Jeanet Conradie, Carl C. Wamser, Abhik Ghosh

**Affiliations:** † Department of Chemistry, UiT—The Arctic University of Norway, N-9037 Tromsø, Norway; ‡ Department of Chemistry, University of the Free State, P.O. Box 339, Bloemfontein 9300, Republic of South Africa; § Department of Chemistry, Portland State University, Portland, Oregon 97207-0751, United States

## Abstract

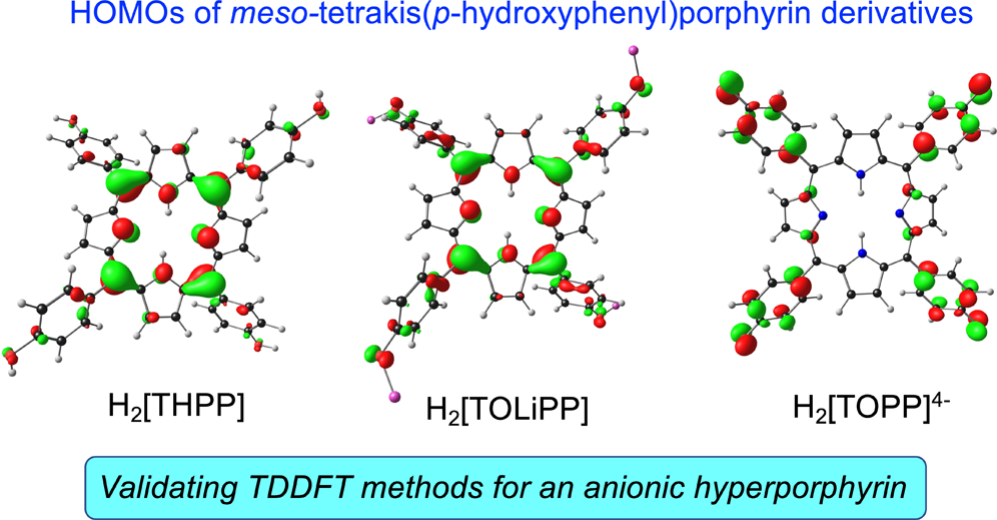

Presented herein is a DFT/TDDFT study of *meso*-tetrakis(4-hydroxyphenyl)porphyrin (H_2_[THPP]) and its *O*-deprotonated tetraanionic form; the latter was modeled as both a free tetraanion and with various counterions. Based on our calculations, the experimentally observed hyperporphyrin spectra are attributed to an admixture of phenol/phenoxide character into the a_2u_-type HOMO of tetraphenylporphyrin. The admixture results in an elevation of the orbital energy of the HOMO in relation to other frontier orbitals, which accounts for the observed spectral redshifts. The calculations underscore differences in the performance of different exchange–correlation functionals. Thus, while the popular hybrid functional B3LYP greatly exaggerates the redshift of the far-red hyperporphyrin band of *O*-deprotonated H_2_[THPP], the range-separated functional CAMY-B3LYP predicts a more moderate redshift. The latter, however, fails to reproduce experimentally observed absorptions in the 550–600 nm range, potentially underscoring the still imperfect modeling of anionic hyperporphyrins.

## Introduction

Martin Gouterman laid the foundations for understanding porphyrin spectroscopy with the four-orbital model, which successfully assigned key features of many porphyrin spectra.^1,2^ He and his co-workers also recognized that many spectra did not quite follow the four-orbital model; notable among these are so-called hyperporphyrins that, by definition, exhibit red-shifted spectral features and/or extra bands relative to normal porphyrins.^3^ They further identified underlying causes of these additional features, attributing them to charge–transfer transitions between the central coordinated atom (which is typically a metal) and the porphyrin ligand, in the form of either metal-to-ligand charge transfer (MLCT) or ligand-to-metal charge transfer (LMCT) transitions.^3^ They later identified charge transfer transitions in metal-free porphyrins involving different parts of the porphyrin itself.^4^ In a perspective on hyperporphyrins, we have described different pathways of charge transfer between *meso*-aryl groups and the heterocyclic core in free-base porphyrins.^5^ In many cases, the hyperporphyrin effect is initiated or amplified by protonation or deprotonation reactions. For example, a particularly dramatic redshift is observed upon protonation of the central nitrogens of *meso*-tetrakis(4-aminophenyl)porphyrin (H_2_[TAPP]), with the lowest-energy band of H_2_[TAPP] shifting from 669 to 813 nm to {H_4_[TAPP]}^2+^ (note that the peripheral amino groups remain unprotonated).^6,7^ The strong delocalization that generates the hyperporphyrin effect can be illustrated with quinone-like resonance forms that show conjugation between the aminophenyl groups and the protonated porphyrin. Gouterman and co-workers performed semiempirical calculations that supported this charge transfer;^8^ more recently, we published a TDDFT study, further refining the spectral assignments for {H_4_[TAPP]}^2+^.^9^

In this study, we have applied the same DFT/TDDFT computational approach to understanding the electronic absorption spectroscopy of *meso*-tetrakis(4-hydroxyphenyl)porphyrin (H_2_[THPP]), which is of significant theoretical interest as well as important for a variety of applications. Thus, Milgrom and co-workers have extensively studied sterically hindered analogues of H_2_[THPP] with *t*-butyl groups flanking the hydroxy groups at the ortho positions. In these cases, the phenols oxidize in air to form stable phenoxy radicals, resulting in hyperporphyrin spectra.^10^ Further oxidation of H_2_[THPP] derivatives leads to oxoporphyrinogens,^11^ which are of interest as catalysts and sensors.^12^ Like H_2_[TAPP],^13,14^ H_2_[THPP] also undergoes oxidative polymerization.^15^ A composite of H_2_[THPP] and cubic Cu_2_O has also been found to exhibit efficient photocatalytic hydrogen evolution.^16^ In many of these applications, the broad visible absorption of the compounds plays a significant role. A DFT/TDDFT study of the electronic absorption spectra of H_2_[THPP] under different conditions using current computational models thus appears to be an eminently worthwhile goal.

The absorption spectra of H_2_[THPP] and its *O*-deprotonated hyperporphyrin form {H_2_[TOPP]}^4–^ have been reported in several different studies.^17−24^ Several of these studies also describe the hyperporphyrin generated by protonation of H_2_[THPP],^17−23^ a hyperporphyrin analogous to that from protonation of H_2_[TAPP]. Briefly, the spectrum of H_2_[THPP] is that of a normal porphyrin with a strong Soret (B) band and four Q bands that fit the Gouterman four-orbital model, while the spectrum of {H_4_[THPP]}^2+^ is that of a mild hyperporphyrin. In a comprehensive study of a series of para-substituted tetraphenylporphyrins (TPPs), H_2_[THPP] shows a distinctive hyperporphyrin redshift upon protonation, unlike TPPs with electron-withdrawing substituents, but not as large as stronger electron-donating substituents such as amino or dimethylamino.^25^ Depending on solvent, the lowest energy Q-band of H_2_[THPP] shifts from 660 nm to about 700 nm upon protonation. For comparison, the data for H_2_[TAPP] are 669 and 813 nm; in addition, the far-red hyperporphyrin band for {H_4_[TAPP]}^2+^ is much stronger than that of {H_4_[THPP]}^2+^.

Of particular interest is a detailed comparative understanding of the cationic hyperporphyrin {H_4_[TAPP]}^2+^ and the anionic hyperporphyrin {H_2_[TOPP]}^4–^. In the case of {H_4_[TAPP]}^2+^, electron density is transferred from the strongly electron-donating *meso*-aminophenyl substituents to the protonated porphyrin core. In {H_2_[TOPP]}^4–^, the porphyrin core is formally neutral, but the meso positions carry strongly electron-donating phenoxide substituents. In spite of the obvious difference in the overall charge situation, the direction of charge transfer is inward in both systems, from the *meso*-aryl groups to the porphyrin core. In a recent Perspective article, we noted that hyperporphyrins exhibiting charge transfer in the opposite direction, i.e., from the porphyrin core to the *meso*-aryl groups, were unknown.^5^ Subsequently such outward charge transfer has been established in anionic metallotriarylcorroles–so-called inverse hypercorroles.^26,27^

**Figure 1:**
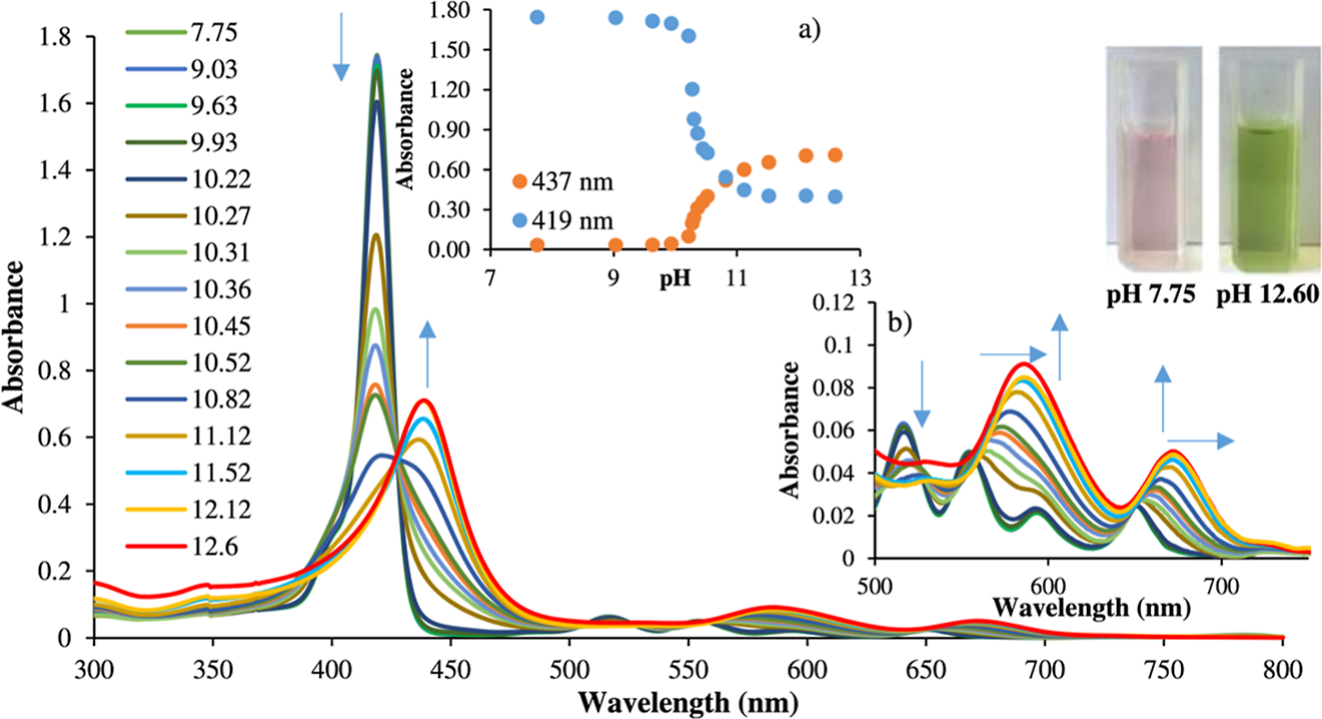
UV–vis absorption spectra of H_2_[THPP] (4.097 × 10^–6^ M) in ethanol as a function of pH, from 7.75 to 12.6, with different concentrations of NaOH. Insets: (a) plot of absorbance at 419 and 453 nm as a function of pH and (b) Q-bands magnification. Reproduced with permission from ref (23) Copyright 2018 Elsevier.

Experimentally, the UV–vis spectra of deprotonated H_2_[THPP] vary substantially with solvent. Thus, the lowest-energy hyperporphyrin band maximum of {H_2_[TOPP]}^4–^ has been reported at a variety of wavelengths between 660 and 705 nm.^17−23^Figure 1 illustrates the simplest case in a protic solvent–ethanol. Recently, Guo et al. have studied the spectra in a wide range of mixed aqueous solutions, noting changes in both the far-red absorption maximum and the C–O stretching frequency. The largest effect was observed in aqueous DMF, where the far-red hyperporphyrin band shifts from 665 nm in pure water to 705 nm in wet DMF with 2% water; for the same solvents, the C–O stretching frequency shifts from 1281 to 1304 cm^–1^.^24^ The increasing spectral redshifts and C–O bond strengths are indicative of greater hyperporphyrin character with decreasing water content, which the authors ascribe to a combination of fewer hydrogen bonding interactions and decreased solvent polarity. In this study, we have simulated different stages of ionization of H_2_[THPP] via a variety of model systems–the fully deprotonated tetraanion {H_2_[TOPP]}^4–^, the same anion with four hydrogen-bonded ethanol molecules {H_2_[TPP]}^4–^·(EtOH)_4_, and the neutral tetralithium, tetrapotassium, and tetrakis(tetramethyl)ammonium salts, H_2_[TOMPP] (M = Li, K, NMe_4_).

## Computational Methods

All molecules were optimized using the scalar-relativistic ZORA (Zeroth Order Regular Approximation to the Dirac equation)^28^ Hamiltonian, the OLYP^29,30^ functional augmented with Grimme’s D3 dispersion correction,^31,32^ all-electron ZORA TZ2P relativistic basis sets, and a *C*_2v_ symmetry constraint. Fine integration grids were used, as were tight criteria for both SCF and geometry cycles, all as implemented in the ADF 2019 program system.^33^ TDDFT calculations were performed on the OLYP-D3 optimized geometries with a variety of hybrid and range-separated functionals; many of the results quoted here are those obtained with the B3LYP^34−37^ and the range-separated^38^ CAMY-B3LYP functionals (which is the Yukawa form of the CAM-B3LYP^39^ functional with *a* = 0.19, *b* = 0.46 and *g* = 0.34, but with the Yukawa potential as the switching function, as opposed to the Coulomb potential attenuated by the complementary error function). In general, we calculated 20 transitions for each system, making sure that the Soret region was adequately covered in each case. All of the above calculations employed the COSMO solvation model (Conductor like Screening Model)^40−42^ with dichloromethane, DMF, and/or ethanol as the solvent.

## Results and Discussion

**Scheme 1:**
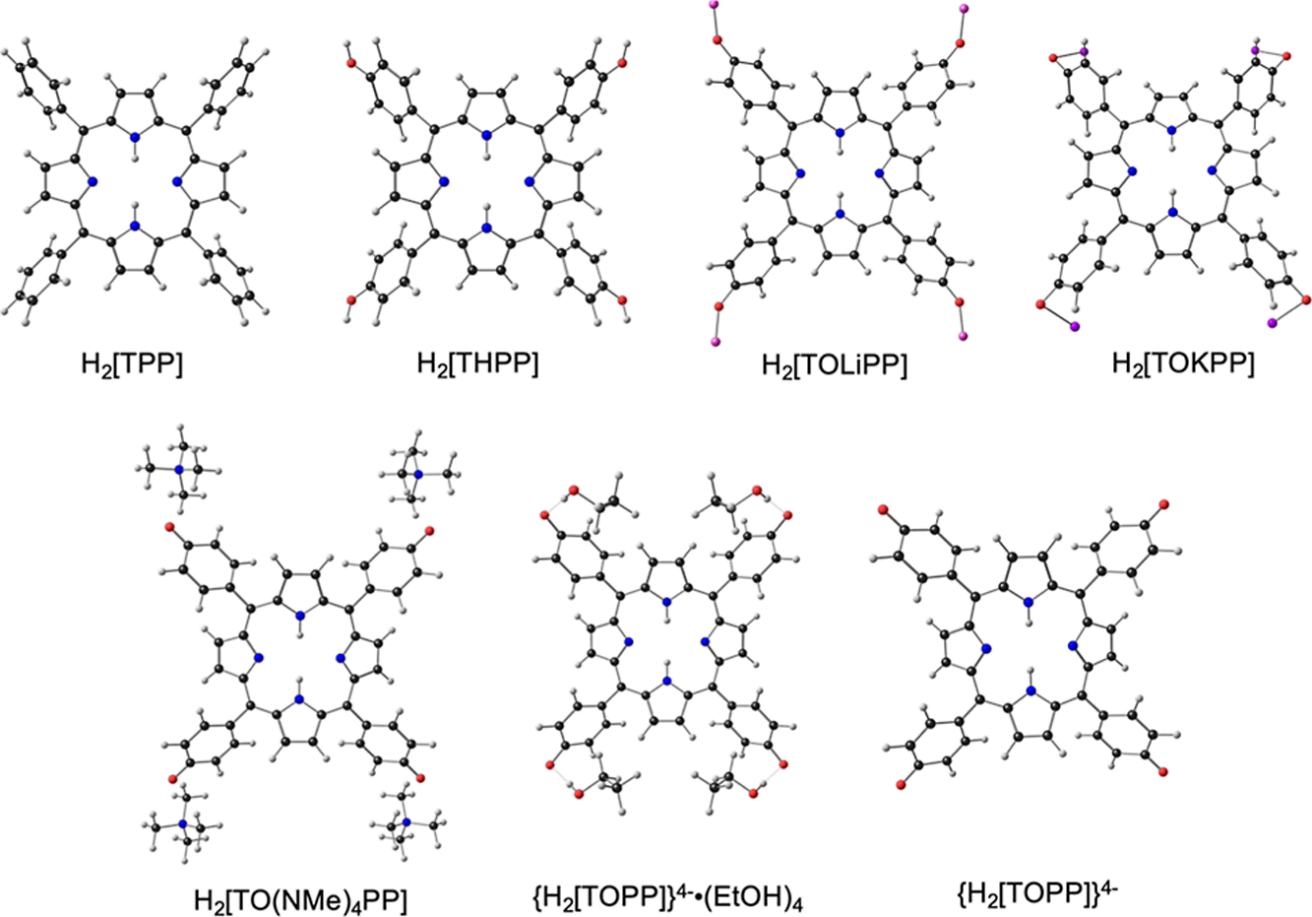
Molecules Studied in This Work: Ball-and-Stick Diagrams are Based OLYP-D3/STO-TZ2P (*C*_2v_) Optimized Geometries

To understand the emergence of hyperporphyrin spectra on going from H_2_[THPP] to {H_2_[TOPP]}^4–^, we chose seven model species for our DFT/TDDFT study (Scheme 1). A wide variety of exchange–correlation functionals were examined; none was fully satisfactory and the present discussion focuses on the popular hybrid functional B3LYP and the range-separated functional CAMY-B3LYP. Together, the calculations appear to provide plausible assignments of key spectral characteristics of the species of interest.

**Figure 2:**
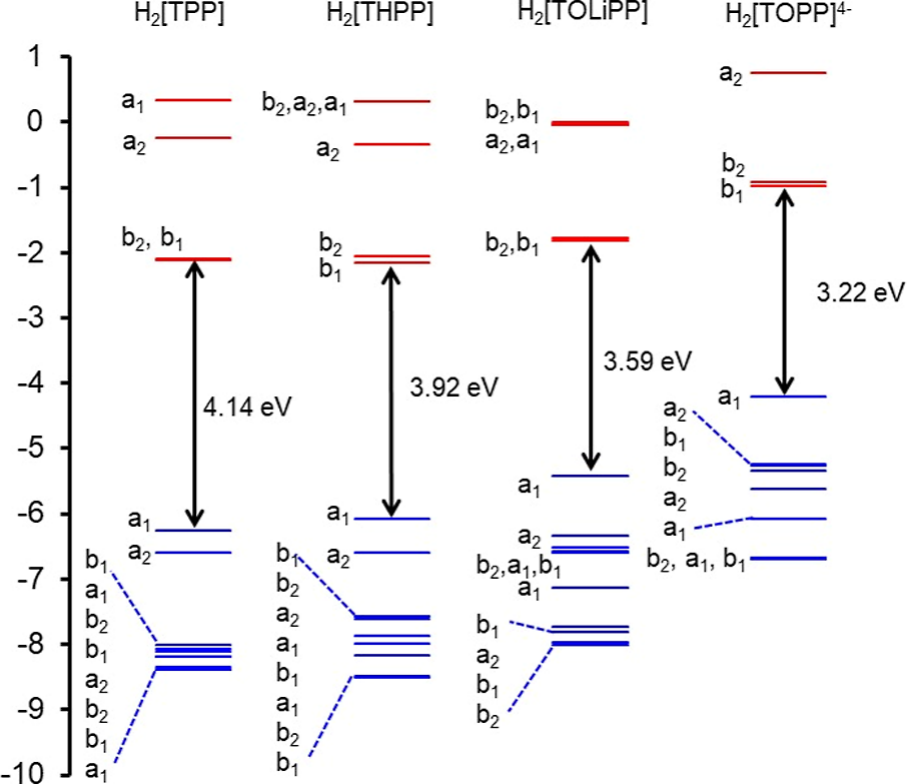
CAMY-B3LYP-D3/STO-TZ2P (COSMO/dichloromethane) frontier MO energy levels for four selected models, along with *C*_2v_ (a_1_, a_2_, b_1_, and b_2_) irreducible representations.

**Figure 3:**
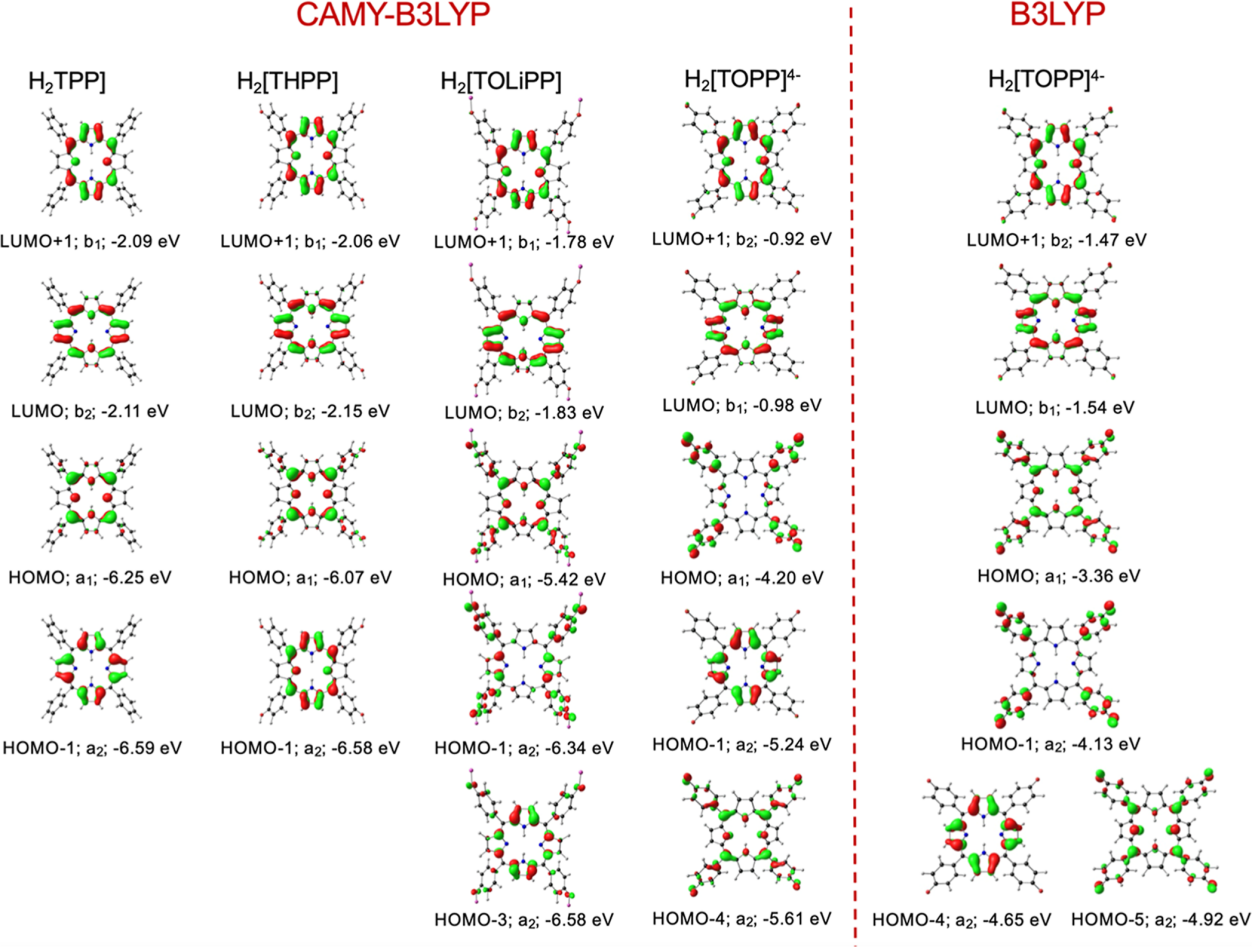
Selected CAMY-B3LYP and B3LYP (COSMO/dichloromethane) frontier MOs, with orbital energies in eV levels for four selected model species, with *C*_2v_ irreducible representations.

The influence of structural perturbation of the *meso*-aryl groups on the porphyrin macrocycle can be readily appreciated from comparative CAMY-B3LYP MO energy level diagrams for a subset of the seven species studied (Figure 2). As expected, unadorned H_2_[TPP] conforms to Gouterman’s four-orbital model, i.e., the two HOMOs and the two LUMOs are energetically well-separated from all other occupied and unoccupied MOs. Also, as expected for porphyrins with electron-donating meso substituents, all three neutral porphyrins studied exhibit a_2u_ HOMOs with large amplitudes at the meso carbons and the central nitrogens. With increasing electron-donating character of the *meso* aryl group, from H_2_[TPP] through H_2_[THPP] and H_2_[TOMPP] (M = Li, K, NMe_4_) to H_2_[TOPP]}^4–^, aryl character mixes into the a_2u_ HOMO, elevating its orbital energy relative to the Gouterman a_1u_ MO and the two LUMOs (Figure 3). This orbital mixing leads to shrinking HOMO–LUMO gaps along the series of compounds studied and for the hyperporphyrin spectra of interest. Not entirely unexpectedly, an extreme scenario arises for the H_2_[TOPP]}^4–^ tetraanion: for the CAMY-B3LYP functional, the HOMO is essentially entirely localized on the phenoxide substituents, with the classic Gouterman a_1u_ and a_2u_ MOs appearing as HOMO – 1 and HOMO – 4, respectively (Figure 3). The dramatic difference in HOMO energy levels between the H_2_[TOMPP] salts and H_2_[TOPP]}^4–^ tetraanion underscores the importance of microsolvation and counterions in determining the HOMO–LUMO gap of peripherally deprotonated H_2_[THPP] under basic conditions, which is consistent with experimental findings.^17−24^ In contrast, changing the solvent in the COSMO scheme (i.e., the dielectric constant) has only a very minor effect on the calculated orbital energies and transition energies (Table S1 and Figure S1).

**Figure 4:**
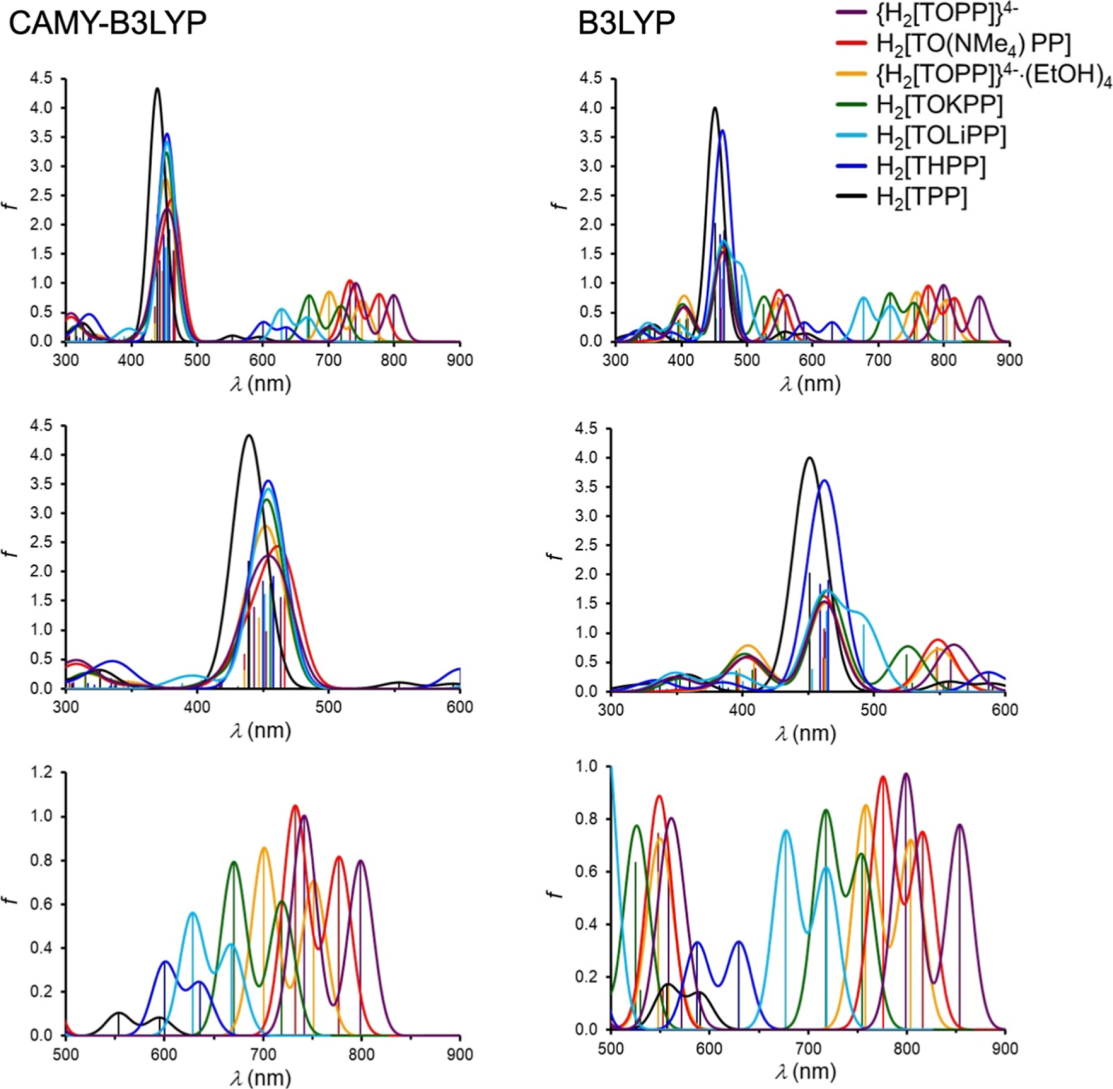
Simulated TDDFT(STO-TZ2P/COSMO) optical spectra in dichloromethane for the B3LYP and CAMY-B3LYP functionals. The vertical lines represent calculated transitions, which have been broadened with Gaussians (fwhm = 30 nm) to generate the simulated spectra.

TDDFT calculations allow a more detailed appreciation of the optical spectra of H_2_[THPP] and its *O*-deprotonated counterpart (Table 1 and Figure 4). Unless otherwise mentioned, the discussion below will focus on CAMY-B3LYP results. For H_2_[TPP], the two Q transitions (ignoring vibrationally excited states that are also observed experimentally) have approximately 2/3 HOMO (generally a_2u_) character and 1/3 HOMO – 1 (a_1u_) character. In more detail, the lower-energy Q transition is approximately 2/3 HOMO-to-LUMO and 1/3 HOMO – 1-to-LUMO + 1, while the higher energy Q-band is approximately 2/3 HOMO-to-LUMO + 1 and 1/3 HOMO – 1-to-LUMO. In H_2_[THPP] and H_2_[TOLiPP], the HOMO (a_2u_) character of the Q transitions is significantly higher, approximately 80% and about 85–90%, respectively. It is this increased HOMO character that accounts for the red-shifted Q bands of H_2_[THPP] and H_2_[TOLiPP] relative to H_2_[TPP]. Moreover, the calculated redshifts relative to H_2_[TPP] are in good accord with those observed experimentally. For the three charge-neutral tetrarylporphyrin systems studied here, the Soret transitions entail a much lower proportion of HOMO character relative to the Q transitions so it makes sense that the spectral redshifts are more muted for the Soret transitions.

**Table 1:** B3LYP and CAMY-B3LYP/STO-TZ2P TDDFT Results, Including Wavelengths (λ), Oscillator Strengths (*f*), MO Compositions, and State Symmetries (as *C*_2v_ Irreps)^a^

		CAMY-B3LYP							B3LYP						
					MO composition							MO composition			
species	peak	E (eV)	λ (nm)	*f*	weight (%)	from	to	state symmetry	*E* (eV)	λ (nm)	*f*	weight (%)	from	to	state symmetry
H_2_[TPP]	Q	2.085	594.7	0.084	69.3	HOMO	LUMO	B_2_	2.101	590.2	0.136	76.3	HOMO	LUMO	B_2_
					28.6	HOMO – 1	LUMO + 1	B_2_				23.0	HOMO – 1	LUMO + 1	B_2_
		2.240	553.6	0.105	67.4	HOMO	LUMO + 1	B_1_	2.227	556.7	0.169	74.7	HOMO	LUMO + 1	B_1_
					31.2	HOMO – 1	LUMO	B_1_				24.9	HOMO – 1	LUMO	B_1_
	Soret	2.822	439.3	2.157	69.6	HOMO – 1	LUMO + 1	B_2_	2.748	451.2	1.985	75.5	HOMO – 1	LUMO + 1	B_2_
					28.0	HOMO	LUMO	B_2_				21.9	HOMO	LUMO	B_2_
		2.824	439.1	2.179	67.2	HOMO – 1	LUMO	B_1_	2.749	451.0	2.019	73.9	HOMO – 1	LUMO	B_1_
					31.1	HOMO	LUMO + 1	B_1_				24.2	HOMO	LUMO + 1	B_1_
{H_2_[THPP]}	Q	1.977	627.2	0.236	78.3	HOMO	LUMO	B_2_	1.969	629.6	0.333	85.5	HOMO	LUMO	B_2_
					19.2	HOMO – 1	LUMO + 1	B_2_				13.7	HOMO – 1	LUMO + 1	B_2_
		2.154	575.5	0.219	74.1	HOMO	LUMO + 1	B_1_	2.112	587.1	0.330	83.0	HOMO	LUMO + 1	B_1_
					24.2	HOMO – 1	LUMO	B_1_				16.5	HOMO – 1	LUMO	B_1_
	Soret	2.751	450.8	2.114	78.7	HOMO – 1	LUMO + 1	B_2_	2.664	465.4	1.896	83.7	HOMO – 1	LUMO + 1	B_2_
					19.2	HOMO	LUMO	B_2_				13.3	HOMO	LUMO	B_2_
		2.780	446.1	2.045	74.0	HOMO – 1	LUMO	B_1_	2.701	459.1	1.832	81.2	HOMO – 1	LUMO	B_1_
					24.2	HOMO	LUMO + 1	B_1_				15.6	HOMO	LUMO + 1	B_1_
H_2_[TOLiPP]	Q	1.802	688.2	0.528	88.0	HOMO	LUMO	B_2_	1.726	718.4	0.613	94.5	HOMO	LUMO	B_2_
					7.1	HOMO – 1	LUMO + 1	B_2_							
		1.935	640.6	0.665	89.4	HOMO	LUMO + 1	B_1_	1.831	677.3	0.753	95.2	HOMO	LUMO + 1	B_1_
					8.2	HOMO – 1	LUMO	B_1_							
	Soret	2.741	452.3	1.974	87.5	HOMO – 1	LUMO + 1	B_2_	2.518	492.4	1.143	95.9	HOMO – 1	LUMO + 1	B_2_
					8.5	HOMO	LUMO	B_2_							
		2.759	449.4	1.616	79.4	HOMO – 1	LUMO	B_1_	2.671	464.2	1.371	78.0	HOMO – 4	LUMO	B_1_
					9.9	HOMO – 3	LUMO	B_1_				12.3	HOMO – 1	LUMO	B_1_
					8.6	HOMO	LUMO + 1	B_1_				5.6	HOMO – 5	LUMO + 1	B_1_
H_2_[TOPP]^4–^	Q	1.552	799.1	0.799	93.5	HOMO	LUMO	B_1_	1.452	853.7	0.779	97.6	HOMO	LUMO	B_1_
		1.672	741.6	1.005	64.6	HOMO – 1	LUMO + 1	B_1_	1.551	799.2	0.973	98.0	HOMO	LUMO + 1	B_2_
					27.8	HOMO – 4	LUMO + 1	B_1_							
									2.170	571.5	0.203	98.7	HOMO – 1	LUMO	B_2_
									2.219	558.6	0.669	98.9	HOMO – 1	LUMO + 1	B_1_
	Soret	2.675	463.5	1.550	94.1	HOMO	LUMO + 1	B_2_	2.679	462.8	1.008	87.4	HOMO – 4	LUMO	B_2_
		2.797	443.2	1.394	76.4	HOMO – 4	LUMO	B_2_				10.1	HOMO – 5	LUMO + 1	B_2_
					17.1	HOMO – 1	LUMO	B_2_	2.687	461.4	0.533	70.6	HOMO – 4	LUMO + 1	B_1_
												26.8	HOMO – 5	LUMO	B_1_

a Contributions >5% are shown for strong Soret and Q peaks.

For the {H_2_[TOPP]}^4–^ tetraanion, our calculations indicate a more unusual scenario. For the CAMY-B3LYP functional, the lowest-energy Q-band is essentially a pure phenoxide-to-LUMO transition, while the higher-energy Q-band has a more conventional four-orbital composition, with comparable contributions from the a_1u_ and a_2u_ MOs. A similar situation also holds for the Soret regime. The lowest-energy Soret transition is essentially a phenoxide-to-LUMO + 1 transition, while the higher-energy transition has a more conventional four-orbital composition involving both the a_1u_ and a_2u_ MOs.

For {H_2_[TOPP]}^4–^, both the B3LYP and CAMY-B3LYP functionals exaggerate the redshift of the far-red hyperporphyrin band (i.e., the lowest energy Q-band) by as much as ∼200 and ∼100 nm, relative to the experimental value (660–705 nm); (Table 1 and Figure 4). Even for the more elaborate model, {H_2_[TOPP]}^4–^·4EtOH, in which each phenoxide oxygen is hydrogen-bonded to an ethanol molecule, the hyperporphyrin band came out overly red-shifted, by ∼130 (B3LYP) and ∼70 nm (CAMY-B3LYP), relative to experiment. These large discrepancies suggest that the tetraanion is a rather extreme representation of *O*-deprotonated H_2_[THPP]. That said, CAMY-B3LYP, as expected, clearly performs better in that it predicts a less extreme redshift of the hyperporphyrin band. On a more speculative note, B3LYP appears to successfully predict transitions of significant intensity (with essentially HOMO – 1-to-LUMO/LUMO + 1 character) in the 550–600 nm range for {H_2_[TOPP]}^4–^ in agreement with experimental spectra, but in sharp contrast to CAMY-B3LYP, which does not indicate any significant absorption whatsoever in this wavelength range.

The differences between the two functionals can be ascribed to a difference in the nature of the HOMO (different degrees of porphyrin-aryl mixing) as well as to orbital reordering for HOMO – 2 to HOMO – 5 (Figure 3). Importantly, other hybrid and range-separated functionals^43^ did not exhibit significant advantages relative to B3LYP and CAMY-B3LYP, respectively. Indeed, CAMY-B3LYP resulted in less extreme redshifts relative to the hybrid functionals ωB97X,^44^ HSE03,^45^ and HSE06^45^ (Table S1 and Figure S2).

Finally, the strength of the hyperporphyrin effect, as measured by the redshift of the lowest-energy absorption band, appears to qualitatively correlate with the OLYP-D3 C_meso_–C_phenyl_ bond distance, which varies as H_2_[TPP] (1.490 Å) > H_2_[THPP] (1.482 Å) ∼ H_2_[TAPP] (1.481 Å) > H_2_[TOLiPP] (1.481 Å) > {H_4_[TPP]}^2+^·2HCO_2_^–^ (1.475 Å) > {H_2_[TOPP]}^4–^ (1.471 Å) > {H_4_[TAPP]}^2+^·2HCO_2_^–^ (1.459 Å). Stated differently, the hyperporphyrin effect appears to increase with increasing quinonoid character of the *meso*-aryl groups, which is expected to be much more important for the all-nitrogen dication {H_4_[TAPP]}^2+^ (which we modeled as a diformate complex in an earlier study^9^) than for the *meso*-phenol/phenoxide-substituted species such as H_2_[THPP], H_2_[TOLiPP], and {H_2_[TOPP]}^4–^.

## Conclusion

In summary, we have attempted to simulate and rationalize the red-shifted hyperporphyrin spectra of *meso*-tetrakis(4-hydroxyphenyl)porphyrin and its *O*-deprotonated tetraanion form using time-dependent density functional theory calculations. The *O*-deprotonated state was modeled in several ways, as a bare tetraanion, with four hydrogen-bonded ethanol molecules, and as salts with a variety of counterions. The spectral redshifts were found to result from an elevation of the porphyrin’s a_2u_-type HOMO energy as a result of admixture of phenol/phenoxide character. Not unexpectedly, the calculations predicted dramatically higher spectral redshifts for the free tetraanion relative to the salts, emphasizing the importance of solvation and counterions in modulating the hyperporphyrin character of the deprotonated state. The calculations also underscored the importance of the choice of the exchange–correlation functional. Thus, while the classic hybrid functional B3LYP was found to greatly exaggerate the redshift of the far-red hyperporphyrin band of the *O*-deprotonated state, the range-separated functional CAMY-B3LYP yielded a more moderate redshift. The latter, however, failed to reproduce experimentally observed transitions in the 550–600 nm range, potentially underscoring the still imperfect modeling of anionic hyperporphyrins.

## Data Availability

The data underlying this study are available in the published article and its Supporting Information.

## Supporting Information Available

Optimized Cartesian coordinates (PDF)
