# Supplementary material for: Understanding Anionic Hyperporphyrins: TDDFT Calculations on Peripherally Deprotonated meso-Tetrakis(4-hydroxyphenyl)porphyrin
Source: J Phys Chem A. 2025 Jan 30;129(6):1591–8. doi: 10.1021/acs.jpca.4c07216 (PMC11831667; doi:10.1021/acs.jpca.4c07216)
Supplement: Supplementary file 1 — jp4c07216_si_001.pdf [file jp4c07216_si_001.pdf]

Supporting Information

Understanding Anionic Hyperporphyrins: TDDFT Calculations on Peripherally Deprotonated *Meso*-tetrakis(4-hydroxyphenyl)porphyrin

Jeanet Conradie,[a,b] Carl C. Wamser,[c] and Abhik Ghosh*[,a]

[a] Department of Chemistry, UiT – The Arctic University of Norway, N-9037 Tromsø, Norway

[b] Department of Chemistry, University of the Free State, P.O. Box 339, Bloemfontein 9300, Republic of South Africa

[c] Department of Chemistry, Portland State University, Portland, Oregon 97207-0751

All-electron OLYP-D3/ZORA-STO-TZ2P Cartesian coordinates (Å) and additional TDDFT data

Contents

1. H_2[TPP], C_{2v} S2
2. H_2[THPP], C_{2v} S3
3. H_2[TOLiTPP], C_{2v} S5
4. H_2[TOKTPP], C_{2v} S6
5. H_2[TOPP]$^{4-}$•(EtOH)$_4$ C_{2v}.. S8
6. H_2[TO(NMe$_4$)TPP], C_{2v} S10
7. H_2[TOPP]$^{4-}$, C_{2v}.. S13
8. Figure S1. TDDFT (CAMY-B3LYP) in DCM, DMF, and EtOH ... S16
9. Figure S2. TDDFT with CAM-B3LYP, ωB97X, and HSE06 ..... S17
10. Table S1. Additional TDDFT data S18

1. H_2[TPP], C_{2v}

C	0.685865000	4.257423000	0.073700000
C	0.685865000	-4.257423000	0.073700000
C	1.132133000	2.898645000	-0.015098000
C	1.132133000	-2.898645000	-0.015098000
C	2.460854000	2.444530000	-0.020735000
C	2.460854000	-2.444530000	-0.020735000
C	2.863717000	1.091347000	-0.032162000
C	2.863717000	-1.091347000	-0.032162000
C	3.519124000	3.492905000	-0.015118000
C	3.519124000	-3.492905000	-0.015118000
C	3.636391000	4.395558000	-1.081735000
C	3.636391000	-4.395558000	-1.081735000
C	4.256624000	0.679282000	-0.148972000
C	4.256624000	-0.679282000	-0.148972000
C	4.413108000	3.597765000	1.060085000
C	4.413108000	-3.597765000	1.060085000
C	4.626220000	5.378642000	-1.076083000
C	4.626220000	-5.378642000	-1.076083000
C	5.400459000	4.583230000	1.069690000
C	5.400459000	-4.583230000	1.069690000
C	5.510922000	5.476933000	0.000985000
C	5.510922000	-5.476933000	0.000985000
C	-0.685865000	4.257423000	0.073700000
C	-0.685865000	-4.257423000	0.073700000
C	-1.132133000	2.898645000	-0.015098000
C	-1.132133000	-2.898645000	-0.015098000
C	-2.460854000	2.444530000	-0.020735000
C	-2.460854000	-2.444530000	-0.020735000
C	-2.863717000	1.091347000	-0.032162000
C	-2.863717000	-1.091347000	-0.032162000
C	-3.519124000	3.492905000	-0.015118000
C	-3.519124000	-3.492905000	-0.015118000
C	-3.636391000	4.395558000	-1.081735000
C	-3.636391000	-4.395558000	-1.081735000
C	-4.256624000	0.679282000	-0.148972000
C	-4.256624000	-0.679282000	-0.148972000
C	-4.413108000	3.597765000	1.060085000
C	-4.413108000	-3.597765000	1.060085000
C	-4.626220000	5.378642000	-1.076083000
C	-4.626220000	-5.378642000	-1.076083000
C	-5.400459000	4.583230000	1.069690000
C	-5.400459000	-4.583230000	1.069690000
C	-5.510922000	5.476933000	0.000985000
C	-5.510922000	-5.476933000	0.000985000
H	0.000000000	1.105799000	-0.081385000
H	0.000000000	-1.105799000	-0.081385000
H	1.343584000	5.109952000	0.142004000
H	1.343584000	-5.109952000	0.142004000
H	2.945859000	4.319471000	-1.916914000
H	2.945859000	-4.319471000	-1.916914000

H	4.326516000	2.901194000	1.889023000
H	4.326516000	-2.901194000	1.889023000
H	4.707449000	6.067450000	-1.914050000
H	4.707449000	-6.067450000	-1.914050000
H	5.102701000	1.344726000	-0.238042000
H	5.102701000	-1.344726000	-0.238042000
H	6.082685000	4.655253000	1.913874000
H	6.082685000	-4.655253000	1.913874000
H	6.280875000	6.245067000	0.007914000
H	6.280875000	-6.245067000	0.007914000
H	-1.343584000	5.109952000	0.142004000
H	-1.343584000	-5.109952000	0.142004000
H	-2.945859000	4.319471000	-1.916914000
H	-2.945859000	-4.319471000	-1.916914000
H	-4.326516000	2.901194000	1.889023000
H	-4.326516000	-2.901194000	1.889023000
H	-4.707449000	6.067450000	-1.914050000
H	-4.707449000	-6.067450000	-1.914050000
H	-5.102701000	1.344726000	-0.238042000
H	-5.102701000	-1.344726000	-0.238042000
H	-6.082685000	4.655253000	1.913874000
H	-6.082685000	-4.655253000	1.913874000
H	-6.280875000	6.245067000	0.007914000
H	-6.280875000	-6.245067000	0.007914000
N	0.000000000	2.117730000	-0.065751000
N	0.000000000	-2.117730000	-0.065751000
N	2.034836000	0.000000000	0.026880000
N	-2.034836000	0.000000000	0.026880000

2. H_2[THPP], C_{2v}

C	0.686933000	4.242798000	0.022151000
C	0.686933000	-4.242798000	0.022151000
C	1.134008000	2.916893000	-0.282439000
C	1.134008000	-2.916893000	-0.282439000
C	2.456754000	2.445047000	-0.342272000
C	2.456754000	-2.445047000	-0.342272000
C	2.825996000	1.090907000	-0.534256000
C	2.825996000	-1.090907000	-0.534256000
C	3.558203000	3.405361000	-0.075925000
C	3.558203000	-3.405361000	-0.075925000
C	3.756488000	4.578552000	-0.817623000
C	3.756488000	-4.578552000	-0.817623000
C	4.195044000	0.679736000	-0.809277000
C	4.195044000	-0.679736000	-0.809277000
C	4.457208000	3.129068000	0.969675000
C	4.457208000	-3.129068000	0.969675000
C	4.819172000	5.437747000	-0.536767000
C	4.819172000	-5.437747000	-0.536767000
C	5.514246000	3.978766000	1.266817000
C	5.514246000	-3.978766000	1.266817000

C	5.702727000	5.138483000	0.507765000
C	5.702727000	-5.138483000	0.507765000
C	-0.686933000	4.242798000	0.022151000
C	-0.686933000	-4.242798000	0.022151000
C	-1.134008000	2.916893000	-0.282439000
C	-1.134008000	-2.916893000	-0.282439000
C	-2.456754000	2.445047000	-0.342272000
C	-2.456754000	-2.445047000	-0.342272000
C	-2.825996000	1.090907000	-0.534256000
C	-2.825996000	-1.090907000	-0.534256000
C	-3.558203000	3.405361000	-0.075925000
C	-3.558203000	-3.405361000	-0.075925000
C	-3.756488000	4.578552000	-0.817623000
C	-3.756488000	-4.578552000	-0.817623000
C	-4.195044000	0.679736000	-0.809277000
C	-4.195044000	-0.679736000	-0.809277000
C	-4.457208000	3.129068000	0.969675000
C	-4.457208000	-3.129068000	0.969675000
C	-4.819172000	5.437747000	-0.536767000
C	-4.819172000	-5.437747000	-0.536767000
C	-5.514246000	3.978766000	1.266817000
C	-5.514246000	-3.978766000	1.266817000
C	-5.702727000	5.138483000	0.507765000
C	-5.702727000	-5.138483000	0.507765000
H	0.000000000	1.152011000	-0.579514000
H	0.000000000	-1.152011000	-0.579514000
H	1.343021000	5.073866000	0.230316000
H	1.343021000	-5.073866000	0.230316000
H	3.083002000	4.813414000	-1.636841000
H	3.083002000	-4.813414000	-1.636841000
H	4.315915000	2.227632000	1.557561000
H	4.315915000	-2.227632000	1.557561000
H	4.970661000	6.335219000	-1.133214000
H	4.970661000	-6.335219000	-1.133214000
H	5.025105000	1.346869000	-0.991274000
H	5.025105000	-1.346869000	-0.991274000
H	6.198274000	3.756594000	2.081361000
H	6.198274000	-3.756594000	2.081361000
H	6.783059000	6.698089000	0.235988000
H	-1.343021000	5.073866000	0.230316000
H	-1.343021000	-5.073866000	0.230316000
H	-3.083002000	4.813414000	-1.636841000
H	-3.083002000	-4.813414000	-1.636841000
H	-4.315915000	2.227632000	1.557561000
H	-4.315915000	-2.227632000	1.557561000
H	-4.970661000	6.335219000	-1.133214000
H	-4.970661000	-6.335219000	-1.133214000
H	-5.025105000	1.346869000	-0.991274000
H	-5.025105000	-1.346869000	-0.991274000
H	-6.198274000	3.756594000	2.081361000
H	-6.198274000	-3.756594000	2.081361000

H	-6.783059000	6.698089000	0.235988000
H	-6.783059000	-6.698089000	0.235988000
N	0.000000000	2.158896000	-0.473259000
N	0.000000000	-2.158896000	-0.473259000
N	2.002049000	0.000000000	-0.423397000
N	-2.002049000	0.000000000	-0.423397000
O	6.767080000	5.936451000	0.834260000
O	6.767080000	-5.936451000	0.834260000
O	-6.767080000	5.936451000	0.834260000
O	-6.767080000	-5.936451000	0.834260000
H	6.783059000	-6.698089000	0.235988000

3. H_2[TOLiTPP], C_{2v}

C	0.687228000	4.172456000	0.401238000
C	0.687228000	-4.172456000	0.401238000
C	1.137002000	2.874279000	0.005602000
C	1.137002000	-2.874279000	0.005602000
C	2.474453000	2.448754000	-0.158503000
C	2.474453000	-2.448754000	-0.158503000
C	2.862749000	1.094897000	-0.326238000
C	2.862749000	-1.094897000	-0.326238000
C	3.391592000	4.685975000	-0.871335000
C	3.391592000	-4.685975000	-0.871335000
C	3.508445000	3.507246000	-0.107820000
C	3.508445000	-3.507246000	-0.107820000
C	4.205718000	0.681833000	-0.696688000
C	4.205718000	-0.681833000	-0.696688000
C	4.290103000	5.736424000	-0.742002000
C	4.290103000	-5.736424000	-0.742002000
C	4.609871000	3.422599000	0.766154000
C	4.609871000	-3.422599000	0.766154000
C	5.366757000	5.686587000	0.187464000
C	5.366757000	-5.686587000	0.187464000
C	5.511032000	4.471299000	0.913131000
C	5.511032000	-4.471299000	0.913131000
C	-0.687228000	4.172456000	0.401238000
C	-0.687228000	-4.172456000	0.401238000
C	-1.137002000	2.874279000	0.005602000
C	-1.137002000	-2.874279000	0.005602000
C	-2.474453000	2.448754000	-0.158503000
C	-2.474453000	-2.448754000	-0.158503000
C	-2.862749000	1.094897000	-0.326238000
C	-2.862749000	-1.094897000	-0.326238000
C	-3.391592000	4.685975000	-0.871335000
C	-3.391592000	-4.685975000	-0.871335000
C	-3.508445000	3.507246000	-0.107820000
C	-3.508445000	-3.507246000	-0.107820000
C	-4.205718000	0.681833000	-0.696688000
C	-4.205718000	-0.681833000	-0.696688000
C	-4.290103000	5.736424000	-0.742002000

C	-4.290103000	-5.736424000	-0.742002000
C	-4.609871000	3.422599000	0.766154000
C	-4.609871000	-3.422599000	0.766154000
C	-5.366757000	5.686587000	0.187464000
C	-5.366757000	-5.686587000	0.187464000
C	-5.511032000	4.471299000	0.913131000
C	-5.511032000	-4.471299000	0.913131000
H	0.000000000	1.125347000	-0.379079000
H	0.000000000	-1.125347000	-0.379079000
H	1.342733000	4.986928000	0.666302000
H	1.342733000	-4.986928000	0.666302000
H	2.567011000	4.772868000	-1.574396000
H	2.567011000	-4.772868000	-1.574396000
H	4.171838000	6.634958000	-1.345914000
H	4.171838000	-6.634958000	-1.345914000
H	4.732423000	2.524212000	1.365985000
H	4.732423000	-2.524212000	1.365985000
H	5.020345000	1.349392000	-0.939366000
H	5.020345000	-1.349392000	-0.939366000
H	6.332371000	4.391015000	1.623142000
H	6.332371000	-4.391015000	1.623142000
H	-1.342733000	4.986928000	0.666302000
H	-1.342733000	-4.986928000	0.666302000
H	-2.567011000	4.772868000	-1.574396000
H	-2.567011000	-4.772868000	-1.574396000
H	-4.171838000	6.634958000	-1.345914000
H	-4.171838000	-6.634958000	-1.345914000
H	-4.732423000	2.524212000	1.365985000
H	-4.732423000	-2.524212000	1.365985000
H	-5.020345000	1.349392000	-0.939366000
H	-5.020345000	-1.349392000	-0.939366000
H	-6.332371000	4.391015000	1.623142000
H	-6.332371000	-4.391015000	1.623142000
N	0.000000000	2.119984000	-0.198109000
N	0.000000000	-2.119984000	-0.198109000
N	2.054450000	0.000000000	-0.140213000
N	-2.054450000	0.000000000	-0.140213000
O	-6.173506000	6.705338000	0.371789000
O	6.173506000	6.705338000	0.371789000
O	6.173506000	-6.705338000	0.371789000
O	-6.173506000	-6.705338000	0.371789000
Li	-6.289762000	-8.399364000	0.628077000
Li	-6.289762000	8.399364000	0.628077000
Li	6.289762000	8.399364000	0.628077000
Li	6.289762000	-8.399364000	0.628077000

4. H_2[TOKTPP], C_{2v}

C	0.688013000	4.209327000	0.300427000
C	0.688013000	-4.209327000	0.300427000
C	1.137107000	2.894684000	-0.031836000

C	1.137107000	-2.894684000	-0.031836000
C	2.473140000	2.454204000	-0.160372000
C	2.473140000	-2.454204000	-0.160372000
C	2.853564000	1.094377000	-0.304588000
C	2.853564000	-1.094377000	-0.304588000
C	3.478049000	4.641449000	-0.928647000
C	3.478049000	-4.641449000	-0.928647000
C	3.531353000	3.484831000	-0.124126000
C	3.531353000	-3.484831000	-0.124126000
C	4.187727000	0.681543000	-0.702006000
C	4.187727000	-0.681543000	-0.702006000
C	4.461362000	5.619719000	-0.876057000
C	4.461362000	-5.619719000	-0.876057000
C	4.641824000	3.361502000	0.736927000
C	4.641824000	-3.361502000	0.736927000
C	5.578755000	5.528062000	0.017583000
C	5.578755000	-5.528062000	0.017583000
C	5.624736000	4.337855000	0.815062000
C	5.624736000	-4.337855000	0.815062000
C	-0.688013000	4.209327000	0.300427000
C	-0.688013000	-4.209327000	0.300427000
C	-1.137107000	2.894684000	-0.031836000
C	-1.137107000	-2.894684000	-0.031836000
C	-2.473140000	2.454204000	-0.160372000
C	-2.473140000	-2.454204000	-0.160372000
C	-2.853564000	1.094377000	-0.304588000
C	-2.853564000	-1.094377000	-0.304588000
C	-3.478049000	4.641449000	-0.928647000
C	-3.478049000	-4.641449000	-0.928647000
C	-3.531353000	3.484831000	-0.124126000
C	-3.531353000	-3.484831000	-0.124126000
C	-4.187727000	0.681543000	-0.702006000
C	-4.187727000	-0.681543000	-0.702006000
C	-4.461362000	5.619719000	-0.876057000
C	-4.461362000	-5.619719000	-0.876057000
C	-4.641824000	3.361502000	0.736927000
C	-4.641824000	-3.361502000	0.736927000
C	-5.578755000	5.528062000	0.017583000
C	-5.578755000	-5.528062000	0.017583000
C	-5.624736000	4.337855000	0.815062000
C	-5.624736000	-4.337855000	0.815062000
H	0.000000000	1.132383000	-0.345490000
H	0.000000000	-1.132383000	-0.345490000
H	1.344540000	5.035412000	0.524510000
H	1.344540000	-5.035412000	0.524510000
H	2.645108000	4.759418000	-1.617941000
H	2.645108000	-4.759418000	-1.617941000
H	4.396532000	6.495725000	-1.520569000
H	4.396532000	-6.495725000	-1.520569000
H	4.709294000	2.481832000	1.372776000
H	4.709294000	-2.481832000	1.372776000

H	4.993746000	1.349170000	-0.970509000
H	4.993746000	-1.349170000	-0.970509000
H	6.454384000	4.225506000	1.511966000
H	6.454384000	-4.225506000	1.511966000
H	-1.344540000	5.035412000	0.524510000
H	-1.344540000	-5.035412000	0.524510000
H	-2.645108000	4.759418000	-1.617941000
H	-2.645108000	-4.759418000	-1.617941000
H	-4.396532000	6.495725000	-1.520569000
H	-4.396532000	-6.495725000	-1.520569000
H	-4.709294000	2.481832000	1.372776000
H	-4.709294000	-2.481832000	1.372776000
H	-4.993746000	1.349170000	-0.970509000
H	-4.993746000	-1.349170000	-0.970509000
H	-6.454384000	4.225506000	1.511966000
H	-6.454384000	-4.225506000	1.511966000
N	0.000000000	2.134359000	-0.211261000
N	0.000000000	-2.134359000	-0.211261000
N	2.049327000	0.000000000	-0.098069000
N	-2.049327000	0.000000000	-0.098069000
O	-6.459800000	6.480302000	0.133390000
O	6.459800000	6.480302000	0.133390000
O	6.459800000	-6.480302000	0.133390000
O	-6.459800000	-6.480302000	0.133390000
K	-5.190352000	-7.855918000	1.959341000
K	-5.190352000	7.855918000	1.959341000
K	5.190352000	7.855918000	1.959341000
K	5.190352000	-7.855918000	1.959341000

5. $H_2[TOPP]^{4-}\bullet(EtOH)_4$ C_{2v}

C	0.688228000	4.170304000	-0.409795000
C	0.688228000	-4.170304000	-0.409795000
C	1.135592000	2.880509000	-0.825971000
C	1.135592000	-2.880509000	-0.825971000
C	2.277535000	6.565428000	2.740969000
C	2.277535000	-6.565428000	2.740969000
C	2.467807000	2.446866000	-0.998298000
C	2.467807000	-2.446866000	-0.998298000
C	2.843407000	1.092860000	-1.183183000
C	2.843407000	-1.092860000	-1.183183000
C	3.093974000	6.531386000	1.461354000
C	3.093974000	-6.531386000	1.461354000
C	3.413637000	4.670855000	-1.688406000
C	3.413637000	-4.670855000	-1.688406000
C	3.517129000	3.473304000	-0.951665000
C	3.517129000	-3.473304000	-0.951665000
C	4.165493000	0.682233000	-1.615877000
C	4.165493000	-0.682233000	-1.615877000
C	4.359071000	5.674746000	-1.583843000
C	4.359071000	-5.674746000	-1.583843000

C	4.654719000	3.319409000	-0.131732000
C	4.654719000	-3.319409000	-0.131732000
C	5.468617000	5.574685000	-0.684471000
C	5.468617000	-5.574685000	-0.684471000
C	5.600848000	4.321288000	-0.004292000
C	5.600848000	-4.321288000	-0.004292000
C	-0.688228000	4.170304000	-0.409795000
C	-0.688228000	-4.170304000	-0.409795000
C	-1.135592000	2.880509000	-0.825971000
C	-1.135592000	-2.880509000	-0.825971000
C	-2.277535000	6.565428000	2.740969000
C	-2.277535000	-6.565428000	2.740969000
C	-2.467807000	2.446866000	-0.998298000
C	-2.467807000	-2.446866000	-0.998298000
C	-2.843407000	1.092860000	-1.183183000
C	-2.843407000	-1.092860000	-1.183183000
C	-3.093974000	6.531386000	1.461354000
C	-3.093974000	-6.531386000	1.461354000
C	-3.413637000	4.670855000	-1.688406000
C	-3.413637000	-4.670855000	-1.688406000
C	-3.517129000	3.473304000	-0.951665000
C	-3.517129000	-3.473304000	-0.951665000
C	-4.165493000	0.682233000	-1.615877000
C	-4.165493000	-0.682233000	-1.615877000
C	-4.359071000	5.674746000	-1.583843000
C	-4.359071000	-5.674746000	-1.583843000
C	-4.654719000	3.319409000	-0.131732000
C	-4.654719000	-3.319409000	-0.131732000
C	-5.468617000	5.574685000	-0.684471000
C	-5.468617000	-5.574685000	-0.684471000
C	-5.600848000	4.321288000	-0.004292000
C	-5.600848000	-4.321288000	-0.004292000
H	0.000000000	1.135764000	-1.222454000
H	0.000000000	-1.135764000	-1.222454000
H	1.345869000	4.976506000	-0.131843000
H	1.345869000	-4.976506000	-0.131843000
H	1.350549000	5.996467000	2.607858000
H	1.350549000	-5.996467000	2.607858000
H	2.016605000	7.596763000	3.007654000
H	2.016605000	-7.596763000	3.007654000
H	2.505093000	6.945400000	0.628762000
H	2.505093000	-6.945400000	0.628762000
H	2.559375000	4.801945000	-2.348366000
H	2.559375000	-4.801945000	-2.348366000
H	2.843709000	6.122404000	3.569000000
H	2.843709000	-6.122404000	3.569000000
H	3.352161000	5.500135000	1.199907000
H	3.352161000	-5.500135000	1.199907000
H	4.244428000	6.600765000	-2.144929000
H	4.244428000	-6.600765000	-2.144929000
H	4.757448000	2.403591000	0.445342000

H	4.757448000	-2.403591000	0.445342000
H	4.908439000	7.088214000	0.923900000
H	4.908439000	-7.088214000	0.923900000
H	4.965637000	1.351210000	-1.898883000
H	4.965637000	-1.351210000	-1.898883000
H	6.436509000	4.205003000	0.684184000
H	6.436509000	-4.205003000	0.684184000
H	-1.345869000	4.976506000	-0.131843000
H	-1.345869000	-4.976506000	-0.131843000
H	-1.350549000	5.996467000	2.607858000
H	-1.350549000	-5.996467000	2.607858000
H	-2.016605000	7.596763000	3.007654000
H	-2.016605000	-7.596763000	3.007654000
H	-2.505093000	6.945400000	0.628762000
H	-2.505093000	-6.945400000	0.628762000
H	-2.559375000	4.801945000	-2.348366000
H	-2.559375000	-4.801945000	-2.348366000
H	-2.843709000	6.122404000	3.569000000
H	-2.843709000	-6.122404000	3.569000000
H	-3.352161000	5.500135000	1.199907000
H	-3.352161000	-5.500135000	1.199907000
H	-4.244428000	6.600765000	-2.144929000
H	-4.244428000	-6.600765000	-2.144929000
H	-4.757448000	2.403591000	0.445342000
H	-4.757448000	-2.403591000	0.445342000
H	-4.908439000	7.088214000	0.923900000
H	-4.908439000	-7.088214000	0.923900000
H	-4.965637000	1.351210000	-1.898883000
H	-4.965637000	-1.351210000	-1.898883000
H	-6.436509000	4.205003000	0.684184000
H	-6.436509000	-4.205003000	0.684184000
N	0.000000000	2.130818000	-1.046052000
N	0.000000000	-2.130818000	-1.046052000
N	2.044208000	0.000000000	-0.960597000
N	-2.044208000	0.000000000	-0.960597000
O	4.286370000	7.302454000	1.644535000
O	4.286370000	-7.302454000	1.644535000
O	6.256688000	6.573434000	-0.452528000
O	6.256688000	-6.573434000	-0.452528000
O	-4.286370000	7.302454000	1.644535000
O	-4.286370000	-7.302454000	1.644535000
O	-6.256688000	6.573434000	-0.452528000
O	-6.256688000	-6.573434000	-0.452528000

6. H_2[TO(NMe_4)TPP], C_{2v}

C	0.689199000	4.172195000	1.491984000
C	0.689199000	-4.172195000	1.491984000
C	1.137691000	2.878991000	1.087622000
C	1.137691000	-2.878991000	1.087622000
C	2.468928000	2.455002000	0.861830000

C	2.468928000	-2.455002000	0.861830000
C	2.840907000	1.095804000	0.671488000
C	2.840907000	-1.095804000	0.671488000
C	3.329146000	4.664507000	0.014352000
C	3.329146000	-4.664507000	0.014352000
C	3.507758000	3.495374000	0.785550000
C	3.507758000	-3.495374000	0.785550000
C	4.121774000	0.682610000	0.129920000
C	4.121774000	-0.682610000	0.129920000
C	4.311601000	5.636348000	-0.092638000
C	4.311601000	-5.636348000	-0.092638000
C	4.743777000	3.367792000	1.457910000
C	4.743777000	-3.367792000	1.457910000
C	5.136608000	9.307868000	-0.036260000
C	5.136608000	-9.307868000	-0.036260000
C	5.574774000	5.528057000	0.585469000
C	5.574774000	-5.528057000	0.585469000
C	5.730918000	4.337086000	1.374586000
C	5.730918000	-4.337086000	1.374586000
C	6.056628000	10.781355000	-1.778348000
C	6.056628000	-10.781355000	-1.778348000
C	6.118615000	8.337214000	-2.062039000
C	6.118615000	-8.337214000	-2.062039000
C	7.555746000	9.409719000	-0.396128000
C	7.555746000	-9.409719000	-0.396128000
C	-0.689199000	4.172195000	1.491984000
C	-0.689199000	-4.172195000	1.491984000
C	-1.137691000	2.878991000	1.087622000
C	-1.137691000	-2.878991000	1.087622000
C	-2.468928000	2.455002000	0.861830000
C	-2.468928000	-2.455002000	0.861830000
C	-2.840907000	1.095804000	0.671488000
C	-2.840907000	-1.095804000	0.671488000
C	-3.329146000	4.664507000	0.014352000
C	-3.329146000	-4.664507000	0.014352000
C	-3.507758000	3.495374000	0.785550000
C	-3.507758000	-3.495374000	0.785550000
C	-4.121774000	0.682610000	0.129920000
C	-4.121774000	-0.682610000	0.129920000
C	-4.311601000	5.636348000	-0.092638000
C	-4.311601000	-5.636348000	-0.092638000
C	-4.743777000	3.367792000	1.457910000
C	-4.743777000	-3.367792000	1.457910000
C	-5.136608000	9.307868000	-0.036260000
C	-5.136608000	-9.307868000	-0.036260000
C	-5.574774000	5.528057000	0.585469000
C	-5.574774000	-5.528057000	0.585469000
C	-5.730918000	4.337086000	1.374586000
C	-5.730918000	-4.337086000	1.374586000
C	-6.056628000	10.781355000	-1.778348000
C	-6.056628000	-10.781355000	-1.778348000

C	-6.118615000	8.337214000	-2.062039000
C	-6.118615000	-8.337214000	-2.062039000
C	-7.555746000	9.409719000	-0.396128000
C	-7.555746000	-9.409719000	-0.396128000
H	0.000000000	1.133811000	0.694928000
H	0.000000000	-1.133811000	0.694928000
H	1.347404000	4.986366000	1.753675000
H	1.347404000	-4.986366000	1.753675000
H	2.397381000	4.786872000	-0.533257000
H	2.397381000	-4.786872000	-0.533257000
H	4.136996000	6.506228000	-0.719845000
H	4.136996000	-6.506228000	-0.719845000
H	4.172180000	9.346782000	-0.543148000
H	4.172180000	-9.346782000	-0.543148000
H	4.893647000	1.349373000	-0.227024000
H	4.893647000	-1.349373000	-0.227024000
H	4.910846000	2.484117000	2.069518000
H	4.910846000	-2.484117000	2.069518000
H	5.076859000	10.798210000	-2.256086000
H	5.076859000	-10.798210000	-2.256086000
H	5.138895000	8.387324000	-2.537760000
H	5.138895000	-8.387324000	-2.537760000
H	5.228301000	10.129725000	0.674201000
H	5.228301000	-10.129725000	0.674201000
H	5.298541000	8.340593000	0.440604000
H	5.298541000	-8.340593000	0.440604000
H	6.136033000	11.579287000	-1.039555000
H	6.136033000	-11.579287000	-1.039555000
H	6.241487000	7.413139000	-1.494247000
H	6.241487000	-7.413139000	-1.494247000
H	6.664533000	4.213160000	1.922459000
H	6.664533000	-4.213160000	1.922459000
H	6.847503000	10.868886000	-2.523769000
H	6.847503000	-10.868886000	-2.523769000
H	6.909063000	8.467788000	-2.801494000
H	6.909063000	-8.467788000	-2.801494000
H	7.608761000	10.228605000	0.321848000
H	7.608761000	-10.228605000	0.321848000
H	7.625034000	8.443054000	0.099277000
H	7.625034000	-8.443054000	0.099277000
H	8.326137000	9.520490000	-1.159813000
H	8.326137000	-9.520490000	-1.159813000
H	-1.347404000	4.986366000	1.753675000
H	-1.347404000	-4.986366000	1.753675000
H	-2.397381000	4.786872000	-0.533257000
H	-2.397381000	-4.786872000	-0.533257000
H	-4.136996000	6.506228000	-0.719845000
H	-4.136996000	-6.506228000	-0.719845000
H	-4.172180000	9.346782000	-0.543148000
H	-4.172180000	-9.346782000	-0.543148000
H	-4.893647000	1.349373000	-0.227024000

H	-4.893647000	-1.349373000	-0.227024000
H	-4.910846000	2.484117000	2.069518000
H	-4.910846000	-2.484117000	2.069518000
H	-5.076859000	10.798210000	-2.256086000
H	-5.076859000	-10.798210000	-2.256086000
H	-5.138895000	8.387324000	-2.537760000
H	-5.138895000	-8.387324000	-2.537760000
H	-5.228301000	10.129725000	0.674201000
H	-5.228301000	-10.129725000	0.674201000
H	-5.298541000	8.340593000	0.440604000
H	-5.298541000	-8.340593000	0.440604000
H	-6.136033000	11.579287000	-1.039555000
H	-6.136033000	-11.579287000	-1.039555000
H	-6.241487000	7.413139000	-1.494247000
H	-6.241487000	-7.413139000	-1.494247000
H	-6.664533000	4.213160000	1.922459000
H	-6.664533000	-4.213160000	1.922459000
H	-6.847503000	10.868886000	-2.523769000
H	-6.847503000	-10.868886000	-2.523769000
H	-6.909063000	8.467788000	-2.801494000
H	-6.909063000	-8.467788000	-2.801494000
H	-7.608761000	10.228605000	0.321848000
H	-7.608761000	-10.228605000	0.321848000
H	-7.625034000	8.443054000	0.099277000
H	-7.625034000	-8.443054000	0.099277000
H	-8.326137000	9.520490000	-1.159813000
H	-8.326137000	-9.520490000	-1.159813000
N	0.000000000	2.124799000	0.890312000
N	0.000000000	-2.124799000	0.890312000
N	2.065324000	0.000000000	0.963487000
N	6.214673000	9.468392000	-1.073948000
N	6.214673000	-9.468392000	-1.073948000
N	-2.065324000	0.000000000	0.963487000
N	-6.214673000	9.468392000	-1.073948000
N	-6.214673000	-9.468392000	-1.073948000
O	6.494592000	6.430024000	0.492714000
O	6.494592000	-6.430024000	0.492714000
O	-6.494592000	6.430024000	0.492714000
O	-6.494592000	-6.430024000	0.492714000

7. $H_2[TOPP]^{4-}$, C_{2v}

C	0.683474000	4.127418000	0.748968000
C	0.683474000	-4.127418000	0.748968000
C	1.096153000	2.841273000	0.223211000
C	1.096153000	-2.841273000	0.223211000
C	2.453319000	2.472462000	0.008108000
C	2.453319000	-2.472462000	0.008108000
C	2.872030000	1.138239000	-0.213705000
C	2.872030000	-1.138239000	-0.213705000
C	3.324673000	4.725017000	-0.704432000

C	3.324673000	-4.725017000	-0.704432000
C	3.491055000	3.514688000	0.006292000
C	3.491055000	-3.514688000	0.006292000
C	4.146109000	0.689313000	-0.671703000
C	4.146109000	-0.689313000	-0.671703000
C	4.309124000	5.699198000	-0.743452000
C	4.309124000	-5.699198000	-0.743452000
C	4.713743000	3.357148000	0.698617000
C	4.713743000	-3.357148000	0.698617000
C	5.561995000	5.558237000	-0.049562000
C	5.561995000	-5.558237000	-0.049562000
C	5.700857000	4.329021000	0.685739000
C	5.700857000	-4.329021000	0.685739000
C	-0.683474000	4.127418000	0.748968000
C	-0.683474000	-4.127418000	0.748968000
C	-1.096153000	2.841273000	0.223211000
C	-1.096153000	-2.841273000	0.223211000
C	-2.453319000	2.472462000	0.008108000
C	-2.453319000	-2.472462000	0.008108000
C	-2.872030000	1.138239000	-0.213705000
C	-2.872030000	-1.138239000	-0.213705000
C	-3.324673000	4.725017000	-0.704432000
C	-3.324673000	-4.725017000	-0.704432000
C	-3.491055000	3.514688000	0.006292000
C	-3.491055000	-3.514688000	0.006292000
C	-4.146109000	0.689313000	-0.671703000
C	-4.146109000	-0.689313000	-0.671703000
C	-4.309124000	5.699198000	-0.743452000
C	-4.309124000	-5.699198000	-0.743452000
C	-4.713743000	3.357148000	0.698617000
C	-4.713743000	-3.357148000	0.698617000
C	-5.561995000	5.558237000	-0.049562000
C	-5.561995000	-5.558237000	-0.049562000
C	-5.700857000	4.329021000	0.685739000
C	-5.700857000	-4.329021000	0.685739000
H	1.140030000	0.000000000	0.235325000
H	1.351950000	4.904477000	1.092093000
H	1.351950000	-4.904477000	1.092093000
H	2.399390000	4.875755000	-1.255862000
H	2.399390000	-4.875755000	-1.255862000
H	4.154573000	6.607938000	-1.325096000
H	4.154573000	-6.607938000	-1.325096000
H	4.867483000	2.449092000	1.277247000
H	4.867483000	-2.449092000	1.277247000
H	4.947562000	1.346719000	-0.971687000
H	4.947562000	-1.346719000	-0.971687000
H	6.620456000	4.180927000	1.251632000
H	6.620456000	-4.180927000	1.251632000
H	-1.140030000	0.000000000	0.235325000
H	-1.351950000	4.904477000	1.092093000
H	-1.351950000	-4.904477000	1.092093000

H	-2.399390000	4.875755000	-1.255862000
H	-2.399390000	-4.875755000	-1.255862000
H	-4.154573000	6.607938000	-1.325096000
H	-4.154573000	-6.607938000	-1.325096000
H	-4.867483000	2.449092000	1.277247000
H	-4.867483000	-2.449092000	1.277247000
H	-4.947562000	1.346719000	-0.971687000
H	-4.947562000	-1.346719000	-0.971687000
H	-6.620456000	4.180927000	1.251632000
H	-6.620456000	-4.180927000	1.251632000
N	0.000000000	2.061662000	-0.057161000
N	0.000000000	-2.061662000	-0.057161000
N	2.128484000	0.000000000	0.023910000
N	-2.128484000	0.000000000	0.023910000
O	6.482402000	6.458055000	-0.082504000
O	6.482402000	-6.458055000	-0.082504000
O	-6.482402000	6.458055000	-0.082504000
O	-6.482402000	-6.458055000	-0.082504000

8. Figure S1. TDDFT CAMY-B3LYP in DCM, DMF, and EtOH

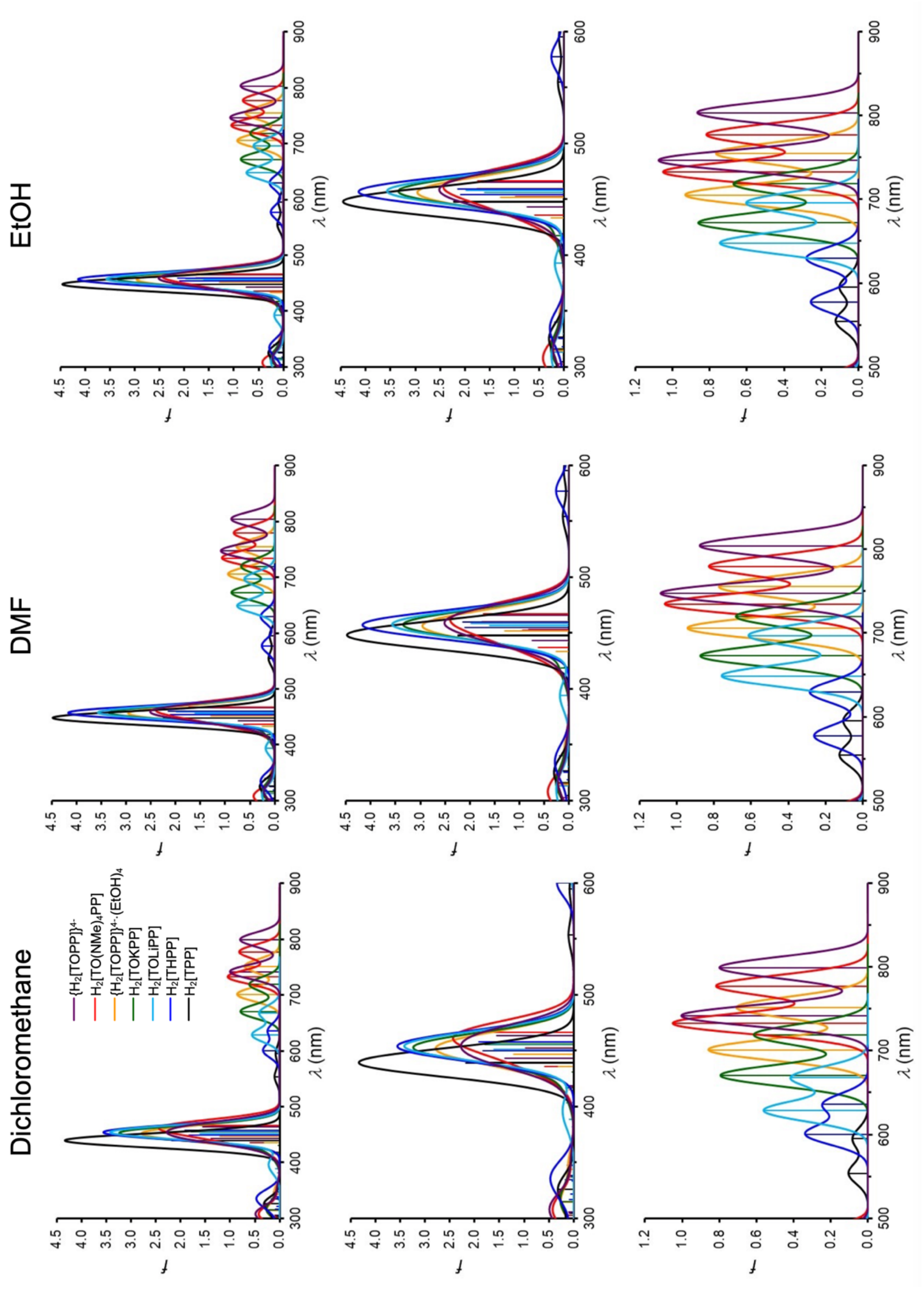

Figure S1. Simulated TDDFT CAMY-B3LYP/STO-TZ2P/COSMO optical spectra in dichloromethane, DMF, and ethanol. The vertical lines represent calculated transitions, which have been broadened with Gaussians (FWHM = 30 nm) to generate the simulated spectra.

9. Figure S2. TDDFT with CAM-B3LYP, ωB97X, and HSE06

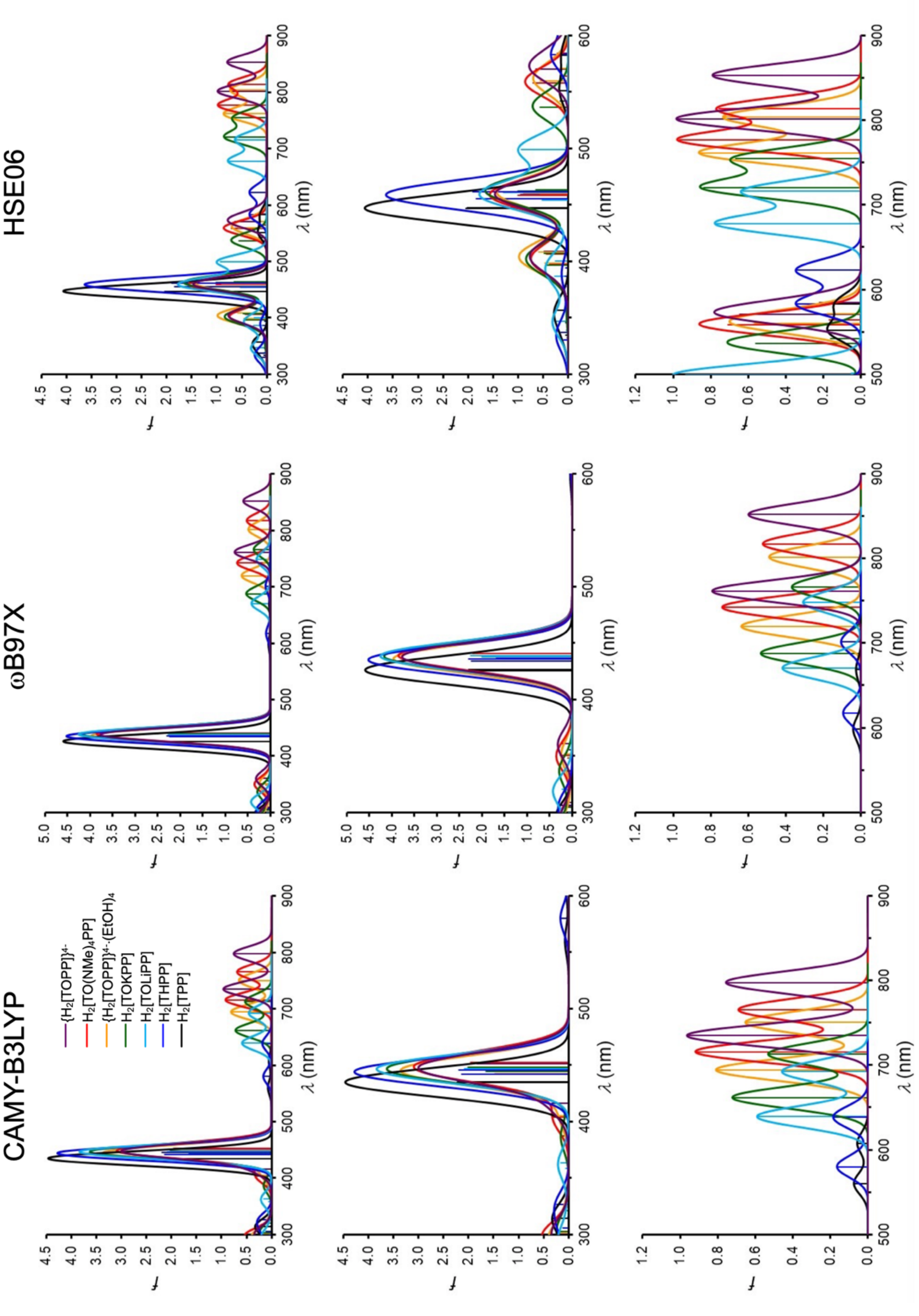

Figure S2. Simulated TDDFT (STO-TZ2P/COSMO/dichloromethane) optical spectra for the CAM-B3LYP, ωB97X, and HSE06 functionals. The vertical lines represent calculated transitions, which have been broadened with Gaussians (FWHM = 30 nm) to generate the simulated spectra.

10. Table S1. Additional TDDFT/STO-TZ2P/COSMO results: absorption maxima (λ in nm) and oscillator strengths (*f*) for different functionals and solvents.

**CAMY-B3LYP (DCM)**	λ	**f**	λ	**f**	λ	**f**	λ	**f**	λ	**f**	λ	**f**
H_2[TPP]	594.74	0.08	553.59	0.11	439.35	2.16	439.07	2.18				
H_2[THPP]	627.24	0.24	575.50	0.22	450.77	2.11	446.07	2.05				
H_2[TOLiPP]	688.16	0.53	640.62	0.66	452.31	1.97	449.42	1.62				
H_2[TOKPP]	711.21	0.60	666.42	0.79	454.88	1.87	448.15	1.50				
$\{H_2[TOPP]\}^{4-}(EtOH)_4$	751.45	0.71	701.04	0.86	457.12	1.68	446.59	1.21				
$H_2[TO(NMe_4)PP]$	765.60	0.74	721.31	0.97	462.41	1.74	447.11	0.95	431.91	0.61		
$\{H_2[TOPP]\}^{4-}$	799.06	0.80	741.56	1.01	463.54	1.55	443.21	1.39				
**CAMY-B3LYP (DMF)**												
H_2[TPP]	595.19	0.10	554.31	0.12	448.24	2.24	447.75	2.24				
H_2[THPP]	629.51	0.29	577.20	0.26	459.54	2.15	454.51	2.10				
H_2[TOLiPP]	696.80	0.61	648.66	0.76	458.47	1.98	456.38	1.60				
H_2[TOKPP]	719.65	0.68	672.45	0.87	459.40	1.88	454.73	1.52				
$\{H_2[TOPP]\}^{4-}(EtOH)_4$	755.13	0.77	705.62	0.94	460.05	1.76	451.90	1.29				
$H_2[TO(NMe_4)PP]$	779.50	0.83	734.46	1.06	467.16	1.73	452.32	0.94	437.26	0.63		
$\{H_2[TOPP]\}^{4-}$	804.18	0.88	747.21	1.09	465.73	1.66	452.70	0.82	443.21	0.74		
**CAMY-B3LYP (EtOH)**												
H_2[TPP]	595.32	0.10	554.57	0.12	447.95	2.23	447.59	2.23				
H_2[THPP]	629.46	0.28	577.30	0.26	459.23	2.14	454.03	2.09				
H_2[TOLiPP]	695.57	0.60	647.59	0.74	458.09	1.98	456.25	1.61				
H_2[TOKPP]	718.38	0.67	671.68	0.86	459.14	1.88	454.44	1.52				
$\{H_2[TOPP]\}^{4-}(EtOH)_4$	754.70	0.77	705.12	0.93	459.80	1.76	451.79	1.29				
$H_2[TO(NMe_4)PP]$	777.17	0.82	732.49	1.05	466.54	1.74	452.12	0.98	435.83	0.59		
$\{H_2[TOPP]\}^{4-}$	803.25	0.87	746.34	1.08	465.30	1.66	452.54	0.80	443.00	0.75		
**B3LYP (DCM)**												
H_2[TPP]	590.25	0.14	556.75	0.17	451.20	1.99	451.00	2.02				
H_2[THPP]	629.58	0.33	587.13	0.33	465.44	1.90	459.10	1.83				
H_2[TOLiPP]	718.39	0.61	677.26	0.75	492.36	1.14	464.18	1.37				
H_2[TOKPP]	754.59	0.66	717.52	0.82	529.35	0.15	524.98	0.63	465.09	1.08	464.30	0.65
$\{H_2[TOPP]\}^{4-}(EtOH)_4$	804.00	0.72	758.32	0.85	558.13	0.16	547.67	0.60	463.79	0.96	462.54	0.56
$H_2[TO(NMe_4)PP]$	816.10	0.74	775.66	0.96	552.79	0.15	547.87	0.75	462.06	1.07	461.37	0.57
$\{H_2[TOPP]\}^{4-}$	853.75	0.78	799.25	0.97	571.49	0.20	558.64	0.67	462.83	1.01	461.42	0.53
**CAM-B3LYP (DCM)**												
H_2[TPP]	607.49	0.06	560.08	0.08	434.86	2.22	434.52	2.24				
H_2[THPP]	638.43	0.19	579.92	0.17	445.76	2.20	441.94	2.13				
H_2[TOLiPP]	692.31	0.46	639.03	0.59	447.10	2.09	445.50	1.76				
H_2[TOKPP]	712.88	0.53	661.58	0.72	448.29	2.02	444.37	1.66				
$\{H_2[TOPP]\}^{4-}(EtOH)_4$	750.32	0.65	694.23	0.81	446.88	1.94	442.58	1.47				
$H_2[TO(NMe_4)PP]$	765.24	0.69	715.72	0.92	452.30	1.96	442.32	1.37				
$\{H_2[TOPP]\}^{4-}$	797.54	0.76	734.76	0.96	451.09	1.84	442.55	1.32				
**HSE06 (DCM)**												
H_2[TPP]	582.52	0.14	551.45	0.17	447.16	2.02	446.91	2.04				
H_2[THPP]	622.68	0.35	582.94	0.35	461.80	1.91	455.59	1.84				
H_2[TOLiPP]	716.02	0.63	677.93	0.77	499.11	0.93	461.01	1.30	454.13	0.53		
H_2[TOKPP]	754.53	0.67	720.47	0.84	541.85	0.16	536.27	0.56	463.38	0.65	461.95	1.01
$\{H_2[TOPP]\}^{4-}(EtOH)_4$	803.85	0.73	761.56	0.86	570.98	0.18	559.71	0.57	461.39	0.53	460.45	0.90
$H_2[TO(NMe_4)PP]$	813.97	0.76	776.74	0.97	563.92	0.17	558.02	0.71	459.79	0.56	458.59	1.01
$\{H_2[TOPP]\}^{4-}$	853.06	0.79	801.59	0.98	584.38	0.22	570.34	0.65	459.69	0.51	459.23	0.96

**WB97X (DCM)**												
H_2[TPP]	668.49	0.03	598.07	0.04	426.30	2.30	425.55	2.30				
H_2[THPP]	701.23	0.11	617.11	0.10	435.88	2.29	434.07	2.24				
H_2[TOLiPP]	748.24	0.31	670.31	0.42	438.60	2.26	437.85	2.00				
H_2[TOKPP]	765.98	0.37	687.69	0.53	438.92	2.24	437.32	1.94				
$\{H_2[TOPP]\}^{4-}(EtOH)_4$	801.67	0.49	719.54	0.64	436.05	2.20	435.96	1.76				
H_2[TO(NMe_4)PP]	817.14	0.52	742.20	0.74	440.92	2.20	435.98	1.73				
$\{H_2[TOPP]\}^{4-}$	851.75	0.60	761.33	0.79	438.64	2.10	436.49	1.69				
